# One health viral metagenomics for pathogen surveillance: robust mNGS workflows for viral detection and genome recovery from swab and tissue specimens

**DOI:** 10.1186/s12866-026-05105-5

**Published:** 2026-05-29

**Authors:** Tristan Russell, Elisa Formiconi, Alison Murphy, Jimmy Hortion, Máire McElroy, Mícheál Casey, Laura Garza Cuartero, John F Mee, Hanne Jahns, Christine Kelly, Joanne Byrne, Eoin R Feeney, Patrick WG Mallon, Virginie W Gautier

**Affiliations:** 1 UCD Centre for Experimental Pathogen Host Research (CEPHR), University College Dublin, Dublin, D04 E1W1 Ireland; 2 UCD School of Medicine, University College Dublin, Dublin, D04 C7X2 Ireland; 3 UCD Conway Institute, University College Dublin, Dublin, D04 E1W1 Ireland; 4 Department of Agriculture, Food, and the Marine Laboratories, Backweston, Celbridge, Kildare, W23 VW2C Ireland; 5 Regional Veterinary Laboratories (RVL) Division, Department of Agriculture, Food and the Marine, Agriculture House, Backweston, Dublin, T12 XD51 Ireland; 6 Teagasc, Moorepark Research Centre, Animal and Bioscience Research Department, Fermoy, P61 P302 Ireland; 7 UCD School of Veterinary Medicine, University College Dublin, Dublin 4, D04 W6F6 Ireland; 8 Department of Infectious Diseases, Mater Misericordiae University Hospital, Dublin, D07 AX57 Ireland; 9 Department of Infectious Diseases, St Vincent’s University Hospital, Dublin, D04 T6F4 Ireland

**Keywords:** Virus Discovery, Disease Surveillance, Pandemic Preparedness

## Abstract

**Background:**

Metagenomic next-generation sequencing (mNGS) is an untargeted approach that enables detection of pathogens directly from samples without prior knowledge of their genetic sequences. In the context of pandemic preparedness and One Health surveillance, there is a pressing need for robust viral mNGS workflows that perform reliably across diverse hosts sample types and pre-analytical conditions.

**Results:**

The study evaluated two shotgun mNGS workflows, one for swabs and one for complex tissue matrices, using a reference repository of clinical and post-mortem samples. The panel comprised swabs and tissue samples positive for 18 DNA and RNA viruses (including 12 species) from nine host species and nine anatomical sites, encompassing a range of transport media, storage temperatures and processing timelines. Quality control metrics were embedded throughout nucleic acid extraction, library preparation and sequencing to monitor performance and support interpretation. Overall, 88.9% of 18 DNA and RNA viruses previously detected by PCR were identified, including from samples with low nucleic acid concentrations (< 1 ng/µl) and variable integrity and purity. The workflows identified viral co-infections that had not been detected by prior targeted testing, as well as Phocid herpesvirus 7 (PHV7) for which no complete reference genome was initially available.

**Conclusions:**

These results demonstrate the feasibility and robustness of the swab and tissue mNGS workflows for virus identification across a range of complex clinical specimens supporting their use in investigations of suspected viral diseases of unknown aetiology and is currently being evaluated for early detection of emerging viral threats at the animal-human interface.

**Supplementary Information:**

The online version contains supplementary material available at 10.1186/s12866-026-05105-5.

## Introduction

Pandemic preparedness is a strategic priority for the European Union driven by the substantial morbidity and mortality associated with recent viral emergence events such as SARS-CoV-2 in humans and Avian influenza virus H5N1 in birds and cattle [1]. Zoonotic spillover between host species has underpinned the major pandemics of the last 50 years including those caused by SARS-CoV-2, HIV and Influenza H1N1 [2].

In response, the EU and World Health Organisation have adopted One Health strategies to pandemic preparedness and response frameworks [1, 3], . Surveillance initiatives, such as One Health – ALL Ireland for European Surveillance (OH-ALLIES) [4], are being established to build capacity for detection of high-risk viral families circulating in animal populations while also enhancing the ability to discover previously unknown viruses in Ireland [5]. Within this context, “Pathogen X” and “Pathogen Y” is a placeholder for hypothetical, unknown human and veterinary agents, respectively, with potential to cause significant health and socio-economic disruption [5–7]. Viruses are considered the most plausible Pathogen X or Pathogen Y agents due to their rapid evolution, host switching capacity and ability to alter their virulence before recognition [8–11].

Viral metagenomic next-generation sequencing (mNGS) enables unbiased detection of both known and novel viruses directly from clinical samples addressing gaps in targeted molecular diagnostics, which rely on prior knowledge of pathogen sequences and are not optimum when viral genomes are highly divergent or co-infections and unexpected pathogens are present [12–14].

mNGS has already made important contributions to the detection and characterisation of emerging viral pathogens, exemplified by the identification of Schmallenberg virus as the aetiological agent of large bovine and ovine abortion storms in Europe in 2011 [15], and the rapid characterisation of SARS-CoV-2 from the first COVID-19 cases in China [8–11], where early sequences facilitated design of PCR assays [16, 17] and vaccines [18–20], phylogenetic analyses to track viral spread [21, 22], and inference of phenotype from genotype [10]. Co-infections can also be identified using mNGS [23]. These strengths distinguish mNGS from targeted methods, yet there are several challenges to mNGS-based pathogen discovery and surveillance. Practical concerns, especially in resource-limited settings, include its cost and requirements for specialist equipment, computing and personnel [24–27]. Issues also exist with reduced analytical sensitivity relative to PCR due to low viral fraction in many samples and the need for careful interpretation to ensure specificity. Recently, technical advances in mNGS have made it more accessible [24–26] and various approaches, including methods of host depletion [28–30] and viral enrichment [31, 32], have been developed to improve the sensitivity of viral mNGS.

**Fig. 1:**
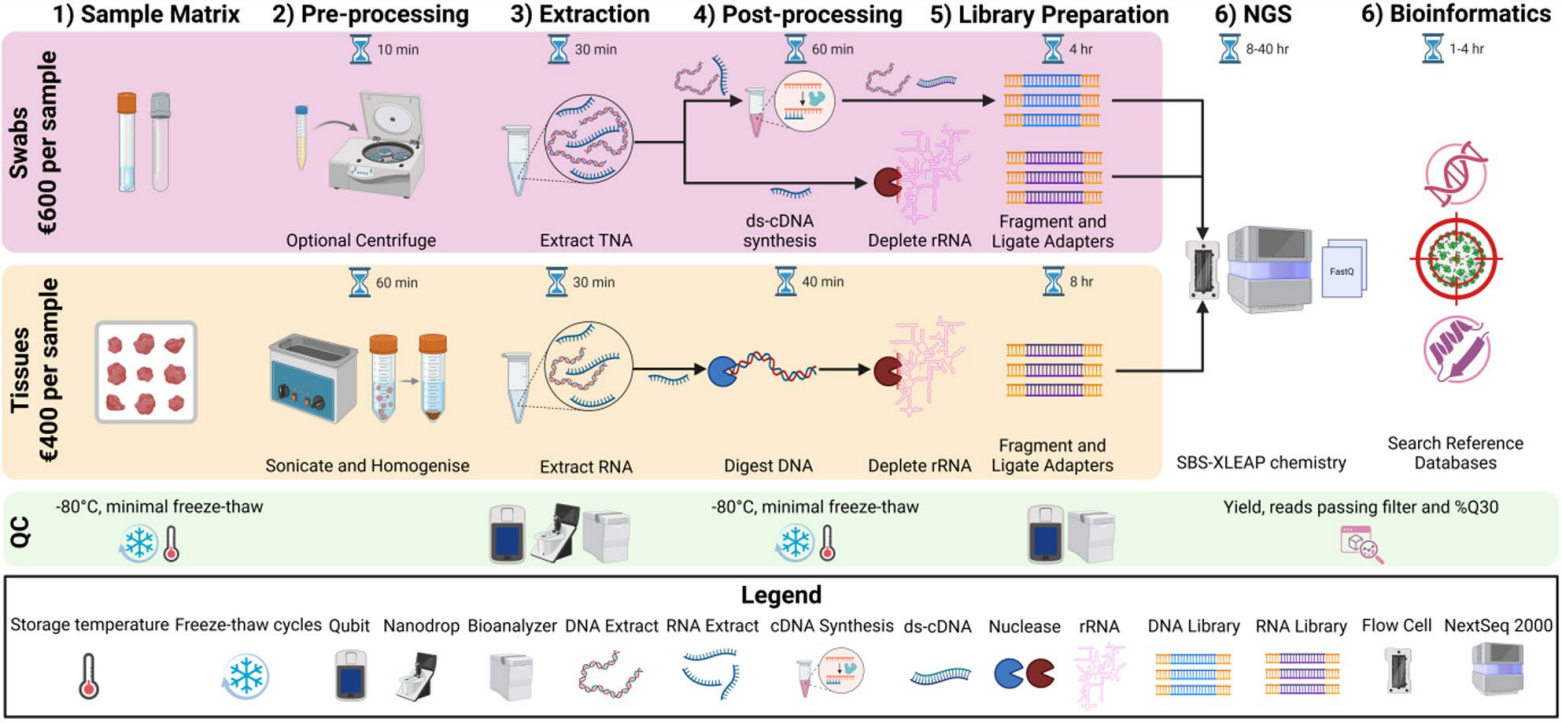
Workflows for shotgun mNGS of clinical samples. The approaches for swab and tissue samples are shown [25, 27, 29, 33–38]. The swab workflow has been designed to detect whole virus particles, so DNA and RNA were analysed by mNGS. Pre-processing nuclease digestions and filtration are included in many swab workflows [25, 29, 33–37], but they were omitted due to the potential for digestion of viral nucleic acids from samples with suboptimal storage and the removal of intracellular viruses [38]. A transcriptomics approach has been applied to detect viral transcripts from tissues. The schematic indicates the critical QC metrics during the workflow, included knowledge of freeze-thaw cycles and storage temperatures and when nucleic acid concentration, purity and integrity is assessed using the Qubit, Nanodrop and Bioanalyzer, respectively. Approximate time to run each step and overall costs per sample are provided. Created with BioRender

There is a particular need for robust and practical mNGS workflows that can operate reliably on the heterogeneous specimens encountered at the animal–human interface spanning both simple swab matrices and complex tissues. Within the OH-ALLIES initiative, two optimised shotgun mNGS workflows were evaluated, one for swab samples and the other for complex tissue matrices (Fig. 1), each integrating comprehensive quality control (QC) measures, from nucleic acid extraction through library preparation and sequencing to bioinformatic analysis, to support robust virus discovery and characterisation [25, 27, 29, 33–38]. The methods detailed here cover the quality metrics, performance for detection of known and unknown viruses, and application of these workflows for consensus genome construction, providing a framework for deployment in One Health surveillance and outbreak investigation.

## Methods

### Reference biospecimen repository

A biospecimen repository of clinical samples positive for known viruses was assembled from five independent sources: the Department of Agriculture, Food and the Marine (DAFM), the Agriculture and Food Development Authority (Teagasc), UCD School of Veterinary Medicine, and the All-Ireland Infectious Disease cohort (AIID). Eight swab and six tissue samples representing nine anatomical sites, 12 viral species and nine host species were included for method development and evaluation (Table 1). All samples from non-human animals were obtained from naturally deceased animals during routine postmortem examinations without the use of anaesthesia or euthanasia.

**Table 1:** Swab and tissue biospecimens used for testing viral mNGS workflows

Sample	Matrix	Host	Anatomical Site	Source	Date of Sampling	Available Details on Animal Condition & Pathology	Storage (°C)^2^	Freeze-thaw cycles	Known Virus (Genome)	Detection
1	Swab (MTM)	*Bos taurus*	Abomasal fluid^1^	Teagasc	02/03/2025	Autolysed Aborted Foetus	-20 & -80^3^	1	SBV (RNA)	qPCR (CT = 32)
2	Swab (UTM)	*Sus scrofa*	Large Intestine	DAFM	2015	Dead, Diarrhoea	-80	≥ 1	Rotavirus A (RNA)	qPCR (CT = 27)
									Rotavirus B (RNA)	qPCR (CT = 31)
3	Swab (UTM)	*Sus scrofa*	Small Intestine	DAFM	2015	Dead, Diarrhoea	-80	≥ 1	Rotavirus A (RNA)	qPCR (CT = 7)
									Rotavirus B (RNA)	qPCR (CT = 17)
									Rotavirus C (RNA)	qPCR (CT = 15)
4	Swab (UTM)	*Sus scrofa*	Faecal	DAFM	2015	Dead, Diarrhoea	-80	≥ 1	Rotavirus A (RNA)	qPCR (CT = 23)
									Rotavirus C (RNA)	qPCR (CT = 22)
5	Swab (Dry)	*Homo sapiens*	Skin	AIID	2025	Live, Skin Poxes	Processed on arrival	0	Mpox IIb (DNA)	qPCR (CT = 27)
6	Swab (Dry)	*Homo sapiens*	Skin	AIID	2025	Live, Skin Poxes	Processed on arrival	0	Mpox Ia (DNA)	qPCR (CT = 24)
7	Swab (Dry)	*Homo sapiens*	Skin	AIID	2022	Live, Skin Poxes	Processed on arrival	0	Mpox IIb (DNA)	qPCR (CT = 26)
8	Swab (UTM)	*Gallus gallus*	Trachea	DAFM	22/04/2024	Dead	-80	≥ 1	MDV (DNA)	qPCR (CT = 33)
9	Tissue	*Elephas maximus*	Heart	UCD Vet	24/07/2024	Dead, systemic haemorrhages, Intestinal ulcers	-20 & -80^3^	1	EEHV1A (DNA)	PCR + ^4^
10	Tissue	*Cervus nippon*	Liver	UCD Vet	19/09/2024	Dead, systemic vasculitis	-20 & -80^3^	1	OHV2 (DNA)	PCR + ^4^
11	Tissue	*Columba livia*	Brain	DAFM	01/07/2020	Dead	-80	≥ 1	PMV1 (RNA)	qPCR (CT = 30)
12	Tissue	*Gallus gallus*	Intestine	DAFM	25/01/2024	Dead	-80	≥ 1	IBV (RNA)	qPCR (CT = 26)
13	Tissue	*Halichoerus grypus*	Brain	UCD Vet	08/11/2022	Dead, stranded, mouth ulcers, septicaemia, umbilical abscess	-20 & -80^3^	2	PHV1 (DNA)	PCR + ^4^
14	Tissue	*Halichoerus grypus*	Gingiva	UCD Vet	17/03/2024	Dead, Stranded, Pneumonia, Septicaemia	-20 & -80^3^	2	PHV7 (DNA)	PCR + ^4^

*Abbreviations*: *EEHV1A* Elephant endotheliotropic herpesvirus, *IBV* Infectious bronchitis virus, *MDV* Marek’s Disease virus, *OHV2* Ovine herpesvirus 2, *MTM* Molecular Transport Media, *PHV1/7* Phocine herpesvirus 1/7, *PMV1* Pigeon paramyxovirus 1, *SBV* Schmallenberg virus; and *UTM* Universal Transport Media

^1^Fetal abomasal fluid comprises swallowed amniotic fluid and gastric secretions – the abomasum is the fourth stomach of a ruminant

^2^ Sample storage prior to extraction, and in some cases, storage of carcasses before postmortem

^3^ Stored at -20 °C when collected and transferred to -80 °C on receipt

^4^ Detected by end-point PCR and confirmed by Sanger sequencing

Swab specimens were collected using flocked swabs and a range of transport and storage conditions. DAFM collected swabs into universal transport medium (COPAN, 330 C), Teagasc collected swabs (COPAN, 552 C) into PrimeStore molecular transport media containing guanidine thiocyanate lysis buffer (Thermo Fisher Scientific, R13905), while AIID dry swabs (COPAN, 552 C) were collected without transport medium. AIID samples were extracted within 2 days of collection to avoid freeze-thaw cycles. DAFM and Teagasc samples were stored at -80 °C and − 20 °C, respectively, until extraction (Table 1).

Tissue biopsies were collected during routine postmortem examinations. Grey seal (*Halichoerus grypus*) carcasses processed by UCD School of Veterinary Medicine were stored at -20 °C prior to necropsies after which tissue biopsies were stored at -80 °C before processing. Sika deer (*Cervus nippon*) and Asian elephant (*Elephas maximus*) tissues collected from fresh animals by UCD Veterinary Medicine were similarly stored at -80 °C before processing. DAFM tissue samples were stored at -80 °C and had undergone at least one freeze-thaw cycle before extraction (Table 1).

Ethical and regulatory oversight was secured through institutional review processes. The UCD Animal Research Ethics Committee granted exemptions for the use of samples collected as part of routine diagnostics or postmortem from DAFM (AREC-E-24-39-Gautier), Teagasc (AREC-E-25-25-Gautier) and UCD School of Veterinary Medicine (AREC-E-22-04-Jahns), and the Human Research Ethics Committee provided approval to work with human clinical samples provided by AIID (306-LS-CSD-25-Mallon).

### Biosafety and risk assessment

All work with infectious material was conducted under appropriate biocontainment with risk assessment determining assignment to Biosafety Level 2 or 3 facilities according to national guidelines. Mpox-positive clinical swabs were handled and processed within a dedicated Biosafety Level 3 laboratory while all remaining animal and human diagnostic or postmortem specimens were manipulated under Biosafety Level 2 conditions using standard operating procedures and engineering controls to minimise exposure risk and prevent environmental release.

### Total Nucleic Acid (TNA) extraction from swabs

Dry Mpox-positive swabs were processed by adding 850 µl Buffer AVL, incubating for 10 min, and using 700 µl lysate for TNA extraction with the QIAamp Viral RNA Mini Kit (Qiagen, 52906) following the manufacturer’s instructions with the sole modification of replacing 6 µl carrier RNA with 6 µl linear acrylamide (5 mg/ml, Thermo Fisher Scientific; AM9520). Linear acrylamide was used instead of carrier RNA to prevent sequencing of carrier RNA. All other swabs were processed by vortexing for 5 s then 300 µl transport media was used for TNA extraction with the Liferiver Viral RNA Isolation Kit (P20211009) on the Liferiver automated extractor platform, following the manufacturer’s instructions except that 6 µl linear acrylamide (5 mg/ml) was substituted for the carrier RNA.

### RNA extraction from tissues

RNA was extracted from tissue samples using the RNeasy Mini Kit (Qiagen, 74106). Tissue was cut on dry-ice into 10–20 mg pieces and transferred into 600 µl Buffer RLT supplemented with 10% β-mercaptoethanol. Samples were sonicated on the high setting of the Biorupter NextGen sonicator (diagenode) for three cycles of 30 s ON and 30 s OFF at 4 °C followed by column-based homogenisation (BioTech, HCR003) at 14,000 x g for 120 s. RNA was purified using the Qiagen RNeasy Mini Kit and residual DNA was digested with the DNA-free DNA Removal Kit (Thermo Fisher Scientific, AM1906) according to manufacturer’s instructions.

### Quality control for nucleic acid extracts

Nucleic acid extract purity and quantity was assessed using the ND-1000 NanoDrop spectrometer (Labtech International). RNA concentrations were determined using the Qubit RNA High Sensitivity RNA Kit (Thermo Fisher Scientific, Q32852) and DNA concentrations using the Qubit DNA High Sensitivity Kits (Thermo Fisher Scientific, Q33230). RNA from tissue extracts was assessed using the Bioanalyzer RNA Nano chip (Agilent, 5067 − 1512) or the Tapestation RNA ScreenTape (Agilent, 5067–5579). Swab extracts with concentrations below the limit of detection of these platforms were not subjected to integrity assessment.

### Double-stranded cDNA synthesis

First-strand cDNA synthesis was performed using SuperScript IV (Thermo Fisher Scientific, 18090050) by combining 11 µl TNA extract with 1 µl 10 mM deoxynucleotides and 1 µl 50 ng/µl random hexamers (Thermo Fisher Scientific, 51709), then incubating at 65 °C for 5 min. Then, 4 µl 5X SSIV Buffer, 1 µl 100 mM DTT, 1 µl RNase OUT (Thermo Fisher Scientific, 100000840) and 1 µl SuperScript IV enzyme were added to reaction mixes, which were incubated at 23 °C for 10 min, 50 °C for 30 min and 80 °C for 10 min.

Second-strand cDNA synthesis was carried out using the Klenow Fragment (Thermo Fisher Scientific, EP0051) by addition of 0.5 µl 10 U per 1 µl Klenow Fragment, 1 µl 10 mM deoxynucleotides, 5 µl Klenow Fragment Buffer and 33.5 µl nuclease-free water to first-strand cDNA synthesis reaction mixes. The reaction was incubated at 37 °C for 30 min, then heat inactivated at 80 °C for 5 min. The final product contained genomic DNA and ds-cDNA.

### cDNA library preparation

cDNA libraries were generated from 500 pg to 100 ng of RNA using the SMART-Seq Total RNA Pico Input with ZapR (Mammalian) rRNA Depletion Kit (Takara Biosciences, 634357) following the manufacturer’s instructions. Fragmentation times were adapted to RNA integrity metrics: 4 min for RIN > 7, 3 min for RIN 5–7, 2 min for RIN 4–5 with DV200 > 50%, and no fragmentation for RIN < 4 with DV200 30–50%. RNA extracts below the limit of detection of the Bioanalyzer and Tapestation were fragmented for 2 min. Following first-strand cDNA synthesis, five PCR cycles were used in first amplification to incorporate adapters and unique dual indexes (Takara Biosciences, 634756), and all purification steps employed NucleoMag NGS Cleanup and Size Select beads (Macherey-Nagel, 744970.50) at a bead: sample ratio of 0.8. Ribosomal RNA was depleted using the ZapR probes provided with the SMART-Seq Kit. A second-round PCR amplification was run for 14 cycles then a final library purification with a bead: sample ratio of 1.0.

### DNA library preparation

DNA libraries were generated from 50 pg to 50 ng of DNA (genomic DNA plus ds-cDNA) using the ThruPLEX DNA-Seq Kit (Takara Biosciences, R400674) according to the manufacturer’s instructions. Sequencing adapters and unique dual indexes were incorporated using PCR with tailed primers. Cycle numbers were adjusted based on DNA input: 8 cycles for 30–50 ng, 9 cycles for 10–30 ng, 10 cycles for 3–10 ng, 11 cycles for 1.5-3 ng, 12 cycles for 0.5–1.5 ng, 14 cycles for 0.2–0.5 ng and 16 cycles for 0.05–0.2 ng. Final DNA libraries were purified using a bead: sample ratio of 1.0.

### Libraries quality control

Library fragment size distribution was assessed using either the Bioanalyzer DNA High Sensitivity Chip (Agilent, 5067 − 4626) or the DNA D1000 TapeStation (Agilent, 5067–5584). Libraries were quantified with the Qubit DNA Broad Range Kit (Thermo Fisher Scientific, Q33260) or Qubit DNA High Sensitivity Kits (Thermo Fisher Scientific, Q33230).

### Illumina next-generation sequencing

Next-Generation Sequencing was performed on the Illumina NextSeq 2000 platform using P2 Cartridges with SBS-XLEAP chemistry and 2 × 150 bp paired-end reads (Illumina, 20100985). Libraries were loaded at 600 pM and spiked with 8% PhiX reference genome (Illumina; FC-110-3002). Resulting FASTQ files were deposited on the Sequence Read Archive (Supplementary Table S1) under BioProject PRJNA1371775, except for datasets derived from human clinical samples, which were excluded in accordance with ethical approval conditions.

### Taxonomic identification with CZID

Metagenomic reads were processed using the Chan Zuckerberg ID (CZID) web-based pipeline (version 8.3) for quality control (QC) and taxonomic assignment [39]. Initial QC steps included trimming low-quality base calls (< Q30) and removing reads with poor quality or low sequence complexity using fastp (0.23.2) [40]. Where appropriate host genomes were available, host-derived reads were filtered by mapping against the host genome using Bowtie2 (2.53) [41] and samtools (1.19) [42]. Minimap2 (2.21-r1071) [43] and Diamond (2.0.15) [44] were used to perform nucleotide and protein searches against NCBI nucleotide (NT) and non-redundant (NR) databases (databases from 06/02/2024), respectively, with an E-value cut-off of less than 10^− 6^ for reported hits. This was followed by *de novo* assembly of classified reads into continuous sequences (contigs) with SPAdes (v3.11.1) [45]. Bowtie2 [41] was then used to map reads back to contigs to determine depth. Contigs were searched against the NCBI NT and NR databases using BLASTN and BLASTX [46], respectively to refine taxonomic assignments. The resulting output was a table containing a list of taxonomic matches with associated read and contig counts for the NT and NR databases.

### Reference-based consensus genomes

For viruses with suitable reference genomes, consensus genomes were generated by trimming low confidence base calls and removing short and low complexity reads with Trimmomatic (0.39) [47]. Quality filtered reads were mapped to the closest reference sequence using Minimap2 (2.30) [43]. Read depth was determined using mosdepth (0.3.3) [48]. Consensus genomes were generated using samtools and bcftools (1.22) [42]. Consensus genomes meeting predefined quality threshold (≥ 60% genome coverage with a minimum read depth 10 [49]) were annotated using VAPiD (v1.6.7) [50] and deposited on GenBank (Supplementary Table S2). For viruses lacking a close reference genome, an alternative workflow was applied: overlapping reads were first assembled *de novo* into contigs using SPAdes (4.2.0) [45] passing the “—meta” flag. Diamond (2.1.10) [44] was then used to translate reads into the six open reading frames and perform BLASTX (2.17) [46] searches of contigs against the NR database. A reference sequence with full-genome assembly and consistently one of the best matches from the BLASTX search was selected for carrying out a reference-based TBLASTX [46] search to identify the coordinates along the genome where contigs mapped. Contig nucleotide sequences were then mapped back to these coordinates to generate a consensus sequence. “N” was assigned to positions with no coverage.

### No-template/negative control and assessment of contamination

A no-template control (NTC) consisting of 300 µl nuclease-free water was processed in parallel with clinical samples, with the tube left open during all handling steps to capture potential laboratory and analytical contaminants. Sequencing reads derived from the NTC were used as background for contaminant profiling and downstream filtering. Potential cross-contamination between specimens was also evaluated by processing and sequencing multiple samples within the same batch and by confirming that virus-specific read assignments remained restricted to the corresponding positive samples.

### Verification of taxonomic hits

The following steps were applied to identify true positive hits. Viral taxa identified in libraries from negative controls were excluded as laboratory or analytical contaminants. Viral taxa annotated as “phages” were not considered further. Candidate taxa in sample libraries were required to have at least two or ten specific and non-overlapping reads matching the NT or NR databases, respectively, with a Bonferroni-corrected E-value <1E-10 to ensure a minimum level of statistical support. Reads and contigs associated with these candidate taxa then underwent BLASTN (for NT-derived hits) or BLASTX (NR-derived hits) searches to validate their taxonomic assignment, confirm specificity and determine their genomic loci. Taxa for which all reads or contigs either failed to return the expected virus in BLAST searches, showed stronger matches to host, bacterial or environmental sequences, or mapped only to a single genomic locus were excluded as false-positive hits. Reference-based consensus genomes were generated for the remaining taxa, and only those with at least 1% genome coverage were retained as true positives. Retained true taxonomic hits were reported with outputs from CZID analysis including the specific reads counts and number and length of contigs, together with reference-based consensus genomes metrics (read counts, genome coverage and the mean read depth) to support their final classification.

## Results

### Quality control overview

mNGS workflows deployable for passive or active surveillance of DNA and RNA viruses derived from a range of sample matrices, host species and anatomical sites were designed and tested. The shotgun mNGS workflows developed within OH-ALLIES were systematically quality controlled from sample acquisition through nucleic acid extraction, library preparation and sequencing to ensure robust viral detection across diverse relevant specimens (Fig. 1).

At each step of the mNGS workflow, quantitative and qualitative QC metrics were recorded to monitor process performance, support troubleshooting and document assay robustness for pathogen discovery applications.

#### Sample acquisition

To reflect realistic conditions of an outbreak scenario, where the source of a novel pathogen is uncertain and sample quality, handling and storage are often suboptimal, mNGS workflows were tested with 14 clinical specimens from the OH-ALLIES Reference Biospecimen Repository including eight swabs and six tissue biopsies collected from nine host species and nine anatomical sites (Table 1). Across this panel, targeted molecular testing had identified 12 viral species belonging to four RNA virus families (*Coronaviridae*, *Paramyxoviridae*, *Peribunyaviridae* and *Sedoreoviridae (the latter two families are segmented viruses)*) and two DNA virus families (*Orthoherpesviridae* and *Poxviridae*), providing a diverse benchmark for mNGS performance (Table 1).

Sample handling and storage conditions were deliberately heterogenous, reflecting limited control over logistics during an emerging infectious event (Table 1). Samples had been stored at -20 °C and − 80 °C, in some cases for up to 10 years, and experienced between zero and at least two pre-extraction freeze-thaw events (Supplementary Table S3). Despite these constraints, known viruses remained detectable by mNGS from Samples 13 and 14 following repeated freeze-thaw cycles and prolonged periods of storage at -20 °C, indicating that the workflows performed well in identifying viruses under suboptimal pre-analytical conditions (Supplementary Table S3).

#### Nucleic acid extraction

**Fig. 2:**
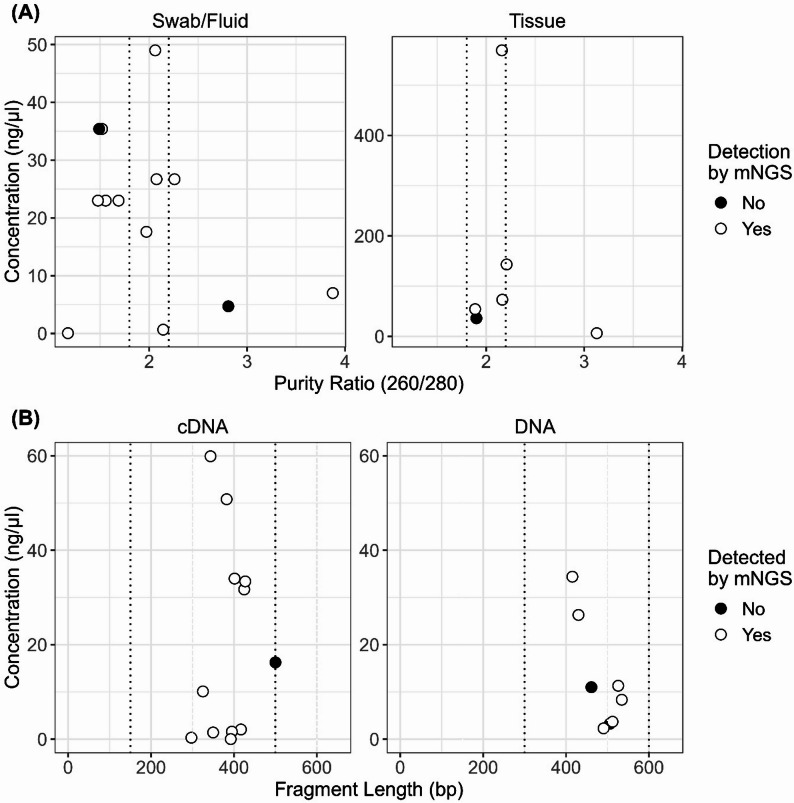
Detection of known viruses by mNGS relative to extract and library measurements. **A** Detection of known viruses relative to extract concentration (y-axis) and purity (x-axis). The 260/280 range of pure nucleic acid (1.8–2.2) is indicated by the dashed vertical lines. **B** Detection of known viruses relative to library concentration (y-axis) and fragment length (x-axis). The recommended fragment length ranges for cDNA and DNA libraries are indicated with dashed vertical lines

Extracts from swabs and tissues encompassed a wide range of DNA and RNA concentrations (below the limit of detection to high nanogram per microliter levels), purity ratios (260/280 and 260/230) and RNA integrity metrics (RIN and DV200) (Supplementary Table S4). These measurements were used to adjust library preparation parameters, with nucleic acid concentrations informing the number of PCR cycles and RIN/DV200 guiding fragmentation times. Known viral targets, including SBV (sample1), Mpox IIb (Sample 5) and MDV (Sample 8), were consistently detected in extracts with low nucleic acid concentration, suboptimal purity and reduced RNA integrity (Supplementary Table S4). Overall, there was no apparent association between nucleic acid extract concentration, integrity or purity and qualitative viral detection by shotgun mNGS, demonstrating the robustness of workflows and their tolerance to degraded or impure input material (Fig. 2A).

#### Library preparation

Library QC focused on concentration and fragment length distribution as both parameters impact data quality and cluster generation on Illumina NGS instruments. All libraries fell within the expected range of fragment length window, (150–500 bp for cDNA libraries and 300–600 bp for DNA libraries) (Fig. 2B), indicating that integrity-guided fragmentation settings were appropriate for cDNA libraries across the tested extract qualities.

Libraries meeting or exceeding the minimum loading concentration (> 600 pM) were generated even from extracts with DNA and RNA concentrations below the limits of detection of high sensitivity fluorometric assays, validating the selected Takara library preparation technologies for low input application. The library concentrations for samples 1 and 5 were below the detection limit of detection, yet the corresponding datasets still yielded qualitative detection of the expected viruses.

#### NGS run performance

Sequencing was carried out on an Illumina NextSeq 2000 instrument using the SBS-XLEAP chemistry, selected for their combined capacity to deliver high throughput and high-quality short read data suited for pathogen discovery applications. Across runs, core instrument metrics such as yield, cluster occupancy and percentage of bases above Q30 exceeded manufacturer specifications (Supplementary Table S5), consistent with high-quality library preparation and optimal run set up.

A target depth of 50 million reads per sample was applied to accommodate the typically low proportion of viral nucleic acid within total sequence output. For swab samples, this target was distributed equally between corresponding DNA and cDNA libraries, with six of eight DNA libraries and five of six cDNA libraries exceeding these targets (Table 2). For tissue samples, 50 million reads per cDNA library was the target and this was achieved for five of six libraries (Table 2). Reads passing filters (post-run QC) and host filtering varied widely between libraries reflecting differences in sample composition and host background (Table 2). Acceptable ranges for total reads and reads passing filters were not established as workflows were designed for compatibility with a range of hosts, anatomical sites and sample integrities, bringing with it variability expected in the number of reads filtered out during QC and host subtraction. Both samples in which the known viruses were not detected by mNGS exceeded 50 million reads with relatively high proportions of passing-filter rates indicating that these metrics alone did not explain occasional false negatives.

**Table 2:** Library-specific metrics for mNGS analysis obtained using CZID and qualitative detection by mNGS. Reads passing filters are those retained following QC and host filtering

Sample	Run	Library Type	Total Reads	Reads Passing Filters	Detection by mNGS
9	1	cDNA	150,000,000	9,968,190 (6.65%)	Yes
10	1	cDNA	141,458,898	11,233,174 (35.76%)	Yes
11	1	cDNA	88,911,054	4,896,870 (3.46%)	No
12	1	cDNA	150,000,000	2,475,474 (2.22%)	Yes
13	1	cDNA	111,705,698	4,357,062 (4.9%)	Yes
14	1	cDNA	31,411,964	3,007,886 (2.01%)	Yes
6	2	cDNA	139,326,058	2,290,160 (1.64%)	Yes
	2	DNA	150,000,000	3,881,426 (2.59%)	Yes
7	2	DNA	60,006,358	111,800 (0.19%)	Yes
8	2	DNA	150,000,000	2,367,062 (1.58%)	Yes
1	2	DNA	4,969,268	3,987,534 (80.24%)	No
	3	cDNA	8,153,070	3,877,596 (47.56%)	Yes
2	3	cDNA	88,164,752	15,326,808 (17.38%)	1 of 2
	3	DNA	46,143,002	2,231,588 (4.84%)	No*
3	3	cDNA	150,000,000	6,399,876 (4.27%)	Yes
	3	DNA	41,962,422	2,252,322 (5.37%)	1 of 3
4	3	cDNA	110,879,828	10,615,516 (9.57%)	1 of 2
	3	DNA	71,164,868	3,439,184 (4.83%)	1 of 2
5	3	cDNA	142,868,726	1,246,670 (66.97%)	Yes
	3	DNA	1,861,622	9,592,780 (6.71%)	Yes

#### Detection of laboratory/analytical contaminants

Laboratory and analytical contaminants were detected running in parallel to clinical samples, mNGS analysis of water maintained in an open tube during swab pre-extraction processing (Supplementary Table S6). Picobirnavirus reads detected in negative controls were also detected in 10 clinical libraries with a high number of reads (Supplementary Table S6). Given the known ubiquity of picobirnaviruses, small non-enveloped bisegmented double-stranded RNA viruses, in laboratories reagents, animals and environments and their appearance across multiple runs, all Picobirnavirus-assigned reads were classified as laboratory contamination, considered artefactual and excluded from downstream analyses [51, 52]. In addition, low-level sporadic signal for Betapapillomavirus 2 and HIV present in the control samples were observed in a subset of clinical libraries (Supplementary Table S6).

#### Overall performance

Comprehensive metadata records captured sample matrix, host species, anatomical site, pre-analytical handling extracts characteristics, library QC metrics and run performance, confirming the workflows were stress tested across a broad spectrum of realistic conditions. Across this range, qualitative detection of known viruses was achieved from both low- and high-quality extracts and libraries, providing evidence that shotgun mNGS workflows are robust for viral pathogen identification in heterogenous outbreak scenarios. Detection of potential laboratory and analytic contaminants underscore the need for routine inclusion of negative controls in future studies, while inclusion of spike-ins as positive controls for the mNGS analysis of cases of unknown aetiology will add an additional layer of quality assurance.

### Identification of known viruses

To develop an NGS-based tool capable of detecting viral causes of infectious disease with unknown aetiology, swab and tissue-specific workflows were evaluated using clinical specimens known to contain diverse viruses. Potential targets include re-emerging viruses with available reference sequences that may escape targeted molecular assays due to phenotypic shifts, sequence variation in regions targeted by PCR or absence from routine testing lists, and truly emerging viruses for which reference sequences might not be available. The latter is covered in the “Identification of Unknown Viruses” section and includes PHV7, which was detected prior to mNGS using PCR and Sanger sequencing of its DNA polymerase gene, yet at the time of analysis no complete reference genome was available.

For viruses with available reference sequences, untargeted shotgun metagenomic NGS data were generated and analysed using the CZID metagenomics pipeline, which aligns non-host reads against the GenBank nucleotide (NT) database for taxonomic identification (Table 3). As detailed in the methods section, only reads specific to the virus taxa were considered and a minimum of two unique reads mapping to distinct genomic loci was required to call a detected virus a true hit consistent with pathogen detection practices [53, 54]. Virus identification was subsequently verified by mapping reads to appropriate references to generate consensus genomes as outlined in the “Reference-based Consensus Genomes” and “Verification of Taxonomic Hits” sections, providing sequence-level confirmation and enabling downstream characterisation.

**Table 3:** Detection of known viruses. Output from the CZID Metagenomics workflow including number of reads matching known virus, proportion of reads matching known virus (reads per million), de novo assembled contigs and Bonferroni corrected E-values. ^1^ The PHV7 reference sequence was unavailable in the GenBank NT database (06/02/2024), but other gammaherpesvirus species were detected in the GenBank NT and NR databases

Sample	Known Virus	Library	CZID Metagenomics Workflow			
			Unique Reads	Reads Per Million	Contigs (Range and Mean)	E-value
1	SBV	cDNA	36	4.4	0	1.92E-81
		DNA	0	0	0	
2	Rotavirus A	cDNA	2	0.1	0	1.02E-61
		DNA	0	0	0	
	Rotavirus B	cDNA	0	0	0	NA
		DNA	0	0	0	
3	Rotavirus A	cDNA	1,735,102	249,386.5	76 (143–3108 and 563.4)	1.21E-165
		DNA	18	7.2	0	
	Rotavirus B	cDNA	78	11.2	1 (303)	1.52E-68
		DNA	0	0	0	
	Rotavirus C	cDNA	44,690	6423.3	17 (155–1858 and 560.5)	1.64E-279
		DNA	0	0	0	
4	Rotavirus A	cDNA	0	0	0	2.89E-67
		DNA	42	11.2	0	
	Rotavirus C	cDNA	2	0.2	0	1.32E-37
		DNA	0	0	0	
5	Mpox IIb	cDNA	1331	715.0	1 (240)	6.72E-272
		DNA	333,126	2,332	107 (230–7429 and 1505.4)	
6	Mpox Ia	cDNA	21	4.5	0	2.91E-84
		DNA	201	11.2	6 (244–315, 290.5)	
7	Mpox IIb	DNA	35,295	588.3	23 (698 − 27,187 and 8285.8)	3.92E-305
8	MDV	DNA	2	0.1	0	1.95E-71
9	EEHV1A	cDNA	11,394	415.8	150 (156–1587 and 422.7)	1.35E-87
10	OHV2	cDNA	26	1.7	0	1.40E-82
11	PMV1	cDNA	0	0	0	NA
12	IBV	cDNA	10	3	0	1.14E-86
13	PHV1	cDNA	2	0.4	0	1.21E-83
14	PHV7^1^	cDNA	0	0	0	NA

#### Known virus detection from swab specimens

For swab specimens, the strategy targeted total nucleic acid to capture both DNA and RNA virus present as whole particles, and therefore both DNA and cDNA libraries were analysed. Across eight swab samples tested containing 12 known viruses, 11 were detected by mNGS with increased qualitative and quantitative detection of RNA and DNA viruses from cDNA and DNA libraries, respectively. Six RNA viruses were detected in cDNA libraries compared to two in DNA libraries and, of the two DNA viruses for which both DNA and cDNA libraries were analysed, there were 333,327 matching reads in DNA libraries compared to 1352 reads in cDNA libraries. One RNA virus (Rotavirus A in Sample 5) was only detected by mNGS analysis of the DNA library. Although across a small sample size, the workflow consistently identified viruses with CTs less than 30 but failed to detect one of the three viruses with a CT greater than 30. Overall, the increased detection of RNA and DNA viruses from cDNA and DNA libraries, respectively, supports the strategy of dual DNA/RNA analysis for swab-based viral surveillance.

#### Known virus detection from tissue specimens

For tissue samples, a transcriptomics approach was prioritised assuming viral transcripts would be present in affected organs. Reference sequences for five known tissue-associated viruses were present in the GenBank NT database and four were detected using mNGS, including three herpesviruses and one coronavirus, demonstrating the detection of viral transcripts from DNA and RNA viruses using this workflow (Table 3). Successful detection of EEHV1A with 11,394 reads and 150 contigs in heart tissue from an elephant with systemic haemorrhages illustrates how pathological examinations can guide optimal samples selection by targeting organs with macroscopically evident lesions for mNGS analysis (Supplementary Figure S1). As three of the five viruses were detected by end-point PCR, the sensitivity of mNGS relative to RT-qPCR for the tissue workflow was not evaluated.

Overall, the 14 specimens used in this evaluation contained 17 known viruses with reference sequences in the NT database of which, 15 (88.2%) were identified including 11 of 12 in swab samples and 4 of 5 in tissue samples (Table 3). The detection rates support the distinct strategies of analysing DNA and RNA for detection of whole virus particles from swab samples and the transcriptomics approach for detecting viral transcripts from tissues.

### Identification of unknown viruses

Shotgun mNGS workflows must be capable of detecting emerging and re-emerging viruses, where there is limited or no prior knowledge of the infectious agent. This spectrum includes “unknown knowns” where reference sequences exist, but the virus has not been tested by routine diagnostics; “known unknowns” where a virus is known to be present, but it lacks a complete reference genome; and “unknown unknowns”, for which neither prior detection nor reference sequences are available. In this study, secondary infections represented unknown knowns, PHV7 served as an example of known unknown, and the workflows were not explicitly challenged with unknown unknowns.

#### Identification of unknown knowns (co-infections)

Co-infections are representative of known unknowns because, although genetic sequences of these viruses are available, their presence in samples was previously unknown. 12 secondary infections were identified across 5 samples (Table 4) applying the criteria of at least two unique reads mapping to different loci of genetic references. Seven enteric viral pathogens were identified in Sample 3 with high read counts matching their respective sequences. Three of these enteric viruses were also observed in Sample 4, although Sapporo virus was deemed a false positive because all reads mapped to a single genomic locus. Molluscum contagiosum virus, Epstein Barr virus (EBV) and Herpes simplex virus 2 (HSV2) were identified as potential co-infections in Samples 5, 6 and 7, respectively, with Molluscum contagiosum virus supported by 1676 reads compared with 44 and 8 reads for HSV2 and EBV, respectively.

**Table 4:** Viral co-infection. Metagenomic workflow details were obtained through analysis with the CZID metagenomics pipeline

Sample	Virus	Library	Metagenomics Workflow			
			Mapped Reads	Reads Per Million	Contigs (Range and Mean)	E-value
3	Porcine astrovius 4	cDNA	352,724	50,697.1	36 (155–6725 and 983.8)	3.79E-300
		DNA	469	188.6	6 (233–3052 and 933.6)	
	Sapporo virus	cDNA	188,694	27,121.0	6 (272–7345 and 1152.8)	1.63E-261
		DNA	820	329.8	3 (450–4189 and 1776.8)	
	Sapelovirus A	cDNA	31,774	4566.9	21 (167–3706 and 688.9)	6.43E-216
		DNA	69	27.8	3 (230–379 and 325.3)	
	Aichivirus C	cDNA	25,729	3698.0	2 (3296–8310 and 5803)	1.22E-168
		DNA	877	352.7	9 (302–2325 and 879)	
	Porcine torovirus	cDNA	18,412	2646.4	6 (306 − 16,744 and 3411.4)	8.43E-296
		DNA	76	30.6	3 (236–380 and 287)	
	Enterovirus G	cDNA	1424	204.7	18 (231–7206 and 719.4)	2.33E-235
		DNA	2	0.8	0	
	Teschovirus A	cDNA	1158	166.4	11 (259–2144 and 667.6)	6.32E-236
		DNA	6	2.4	0	
4	Aichivirus C	DNA	26	6.9	1 (470)	1.22E-94
	Sapelovirus A	DNA	4	1.1	0	8.39E-75
	Sapporo virus	DNA	2	0.5	0	6.59E-67
5	Molluscum contagiosum virus	DNA	1676	11.7	2 (241–267 and 254)	3.86E-72
6	EBV	DNA	8	0.4	0	2.75E-81
7	HSV2	DNA	44	0.7	1 (259)	8.87E-72

#### Identification of known unknowns (PHV7)

Known unknowns had previously been identified in samples, but complete genome reference sequences were unavailable in the NT database when analysis was carried out. PHV7 exemplified this category because a panherpesvirus PCR with Sanger sequencing had confirmed its presence, yet its complete genome sequence was unavailable. Viruses diverge more at the nucleotide level and share more similarity at the amino acid level. As such, an amino acid search was carried out to identify PHV7 by translating reads in all six open reading frames and searching translated sequences against the NR database. Hits to members of the *Gammaherpesvirus* genus in the NR database that did not match to the NT database, were interpreted as specific evidence for PHV7.

PHV7 was not identified when mNGS reads were searched against the NT database, although 18 reads mapped to other species belonging to the *Gammaherpesvirus* subfamily. In contrast, BLASTX searches against the NR database identified 4,050 reads matching members of the *Gammaherpesvirus* subfamily with a Bonferroni corrected E-value of 2.25E-20. On average, the 14 *de novo* assembled contigs (234–931 bp in length) matched reference sequences with 45.14–81.05% amino acid identity. A partial consensus genome sequence was assembled from the metatranscriptomic data, which covered approximately 4.17% of the PHV7 genome (Supplementary Figure S2).

### Conclusion

These shotgun mNGS workflows are intended for deployment in scenarios where prior knowledge of circulating viruses and reference sequences may be incomplete or absent. Detection of 12 secondary infections demonstrates the capacity to identify unknown knowns, while recovery of *Gammaherpesvirus* genus sequences corresponding to PHV7 illustrate that the mNGS workflows can also detect and partially characterise known unknowns. At this stage, workflows were not explicitly tested with “unknown unknowns”, but the data supports their feasibility for detection of novel viruses with at least 45% amino acid identity but detection of viruses with less than 45% identity was not tested.

### Reference-based consensus genomes

Compared to targeted approaches such as PCR and ELISA, shotgun mNGS generates rich sequence data, which can be used to generate partial or complete consensus genomes. These reference-based genomes support the design of targeted diagnostics and mRNA vaccines, enable phylogenetic analysis to track transmission and evolution, and allow inference of phenotypic features from genotype. Reference-based consensus genomes were also used to verify viruses identified by mNGS by assessing read distribution across reference genomes. Consensus genomes were assembled by mapping reads to reference sequences with Minimap2 [43], generating consensus genomes with samtools and bcftools [42] and measuring read depth with mosdepth [48] (Table 5). Typically, more reads mapped to viral sequences during this targeted re-alignment step (Table 5) than were counted by the CZID metagenomic pipeline (Table 4), reflecting inclusion of non-specific reads and more efficient read alignment when the reference genome is provided.

**Table 5:** 1X consensus genomes. Viruses detected with the metagenomic workflow were mapped against reference sequences using Minimap2. The total genome metrics are shown for segmented viruses with a detailed breakdown between segments in Supplementary Table S7

Sample	Known Virus	Library	Consensus Genome			
			Ref. Accession	No. of Reads	1X Coverage (%)	Mean Depth
1	SBV	cDNA	Table S5	36	1.7	0.44
2	Rotavirus A	cDNA	Table S5	18	3.4	0.09
3	Rotavirus A	cDNA	Table S5	24,916,386	85.3	104,903.8
		DNA	Table S5	133	14.59	0.65
	Rotavirus B	cDNA	Table S5	362	4.1	4.99
	Rotavirus C	cDNA	Table S5	22,446,004	80.5	95,253.5
		DNA	Table S5	341	16.7	2.19
	Porcine astrovirus 4	cDNA	KX060808.1	1,463,725	90.2	30,307.1
	Sapporo virus	cDNA	MK962340.1	818,991	93.2	15,239.8
	Sapelovirus A	cDNA	MN836683.1	230,101	92.4	4138.12
	Aichivirus C	cDNA	LC210609.1	342,572	100	5861.0
	Porcine torovirus	cDNA	LT900503.1	429,422	98.2	2161.81
	Enterovirus G	cDNA	MF782664.1	9514	83.4	163.1
	Teschovirus A	cDNA	JQ429405.1	6715	62.9	127.08
4	Rotavirus A	DNA	Table S5	341	16.7	2.19
	Rotavirus C	cDNA	Table S5	5	1.5	0.02
	Aichivirus C	DNA	LC210609.1	204	58.5	3.3
	Sapelovirus A	DNA	MN836683.1	6	3.8	0.11
	Sapporo virus	DNA	MK962340.1	22	12.6	0.38
5	Mpox IIb	cDNA	LC852831.1	2438	0.94	1.21
		DNA	LC852831.1	371,820	93.4	261.5
	Molluscum contagiosum virus	DNA	MH320554.1	366,593	4.44	60.8
6	Mpox Ia	cDNA	LC852831.1	1036	18.19	0.66
		DNA	OZ254457.1	4234	52.9	1.8
	EBV	DNA	NC_007605.1	243	1.54	0.066
7	Mpox IIb	DNA	LC852831.1	39,631	99.9	23.2
	HSV2	DNA	KY922721.1	3225	2.91	0.67
8	MDV	DNA	NC_075702.1	348,573	1.2	190.5
9	EEHV1A	cDNA	KC618527.1	6,666,011	77.5	312.3
10	OHV2	cDNA	PV231823.1	869	6.8	0.46
12	IBV	cDNA	ON350837.1	27,549	21.8	31.1
13	PHV1	cDNA	OK032545.1	103	4.4	0.095

#### Reference-based consensus genomes for verification of virus hits

**Fig. 3:**
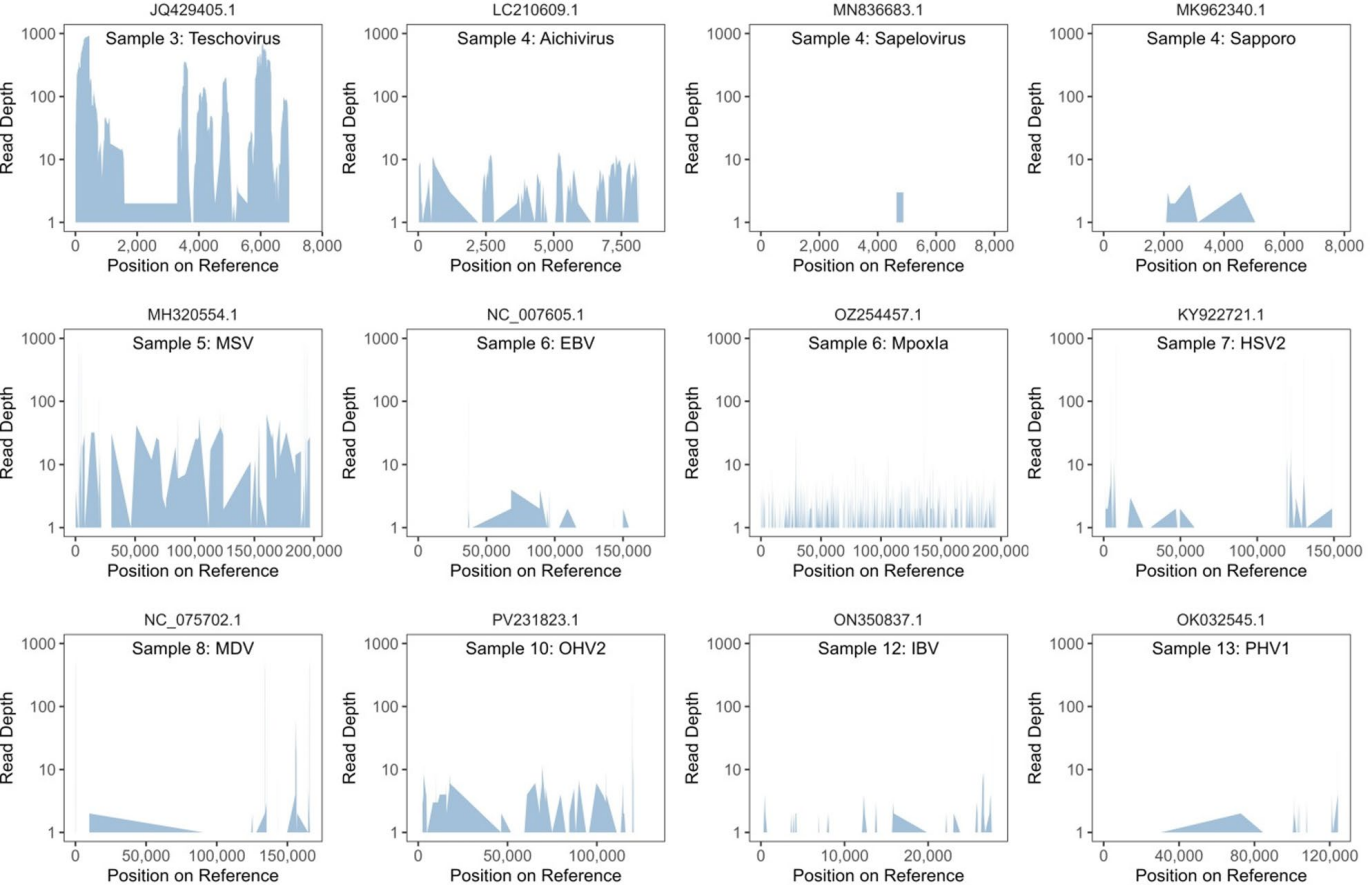
Coverage plots of non-segmented viruses detected by mNGS. Reads were aligned to references using Minimap2 and depth of coverage was determined using mosdepth

Genome-wide coverage profiles were used to distinguish true viral detections from false positive. For most non-segmented virus, coverage plots showed reads mapped to multiple regions of the genome consistent with genuine infections (Fig. 3). In contrast, all Sapelovirus A reads from sample 4, mapped to a single location along its reference genome (Fig. 3), failing the criteria of at least two unique reads mapping to distinct loci, and this signal was therefore classified as false positive.

**Fig. 4:**
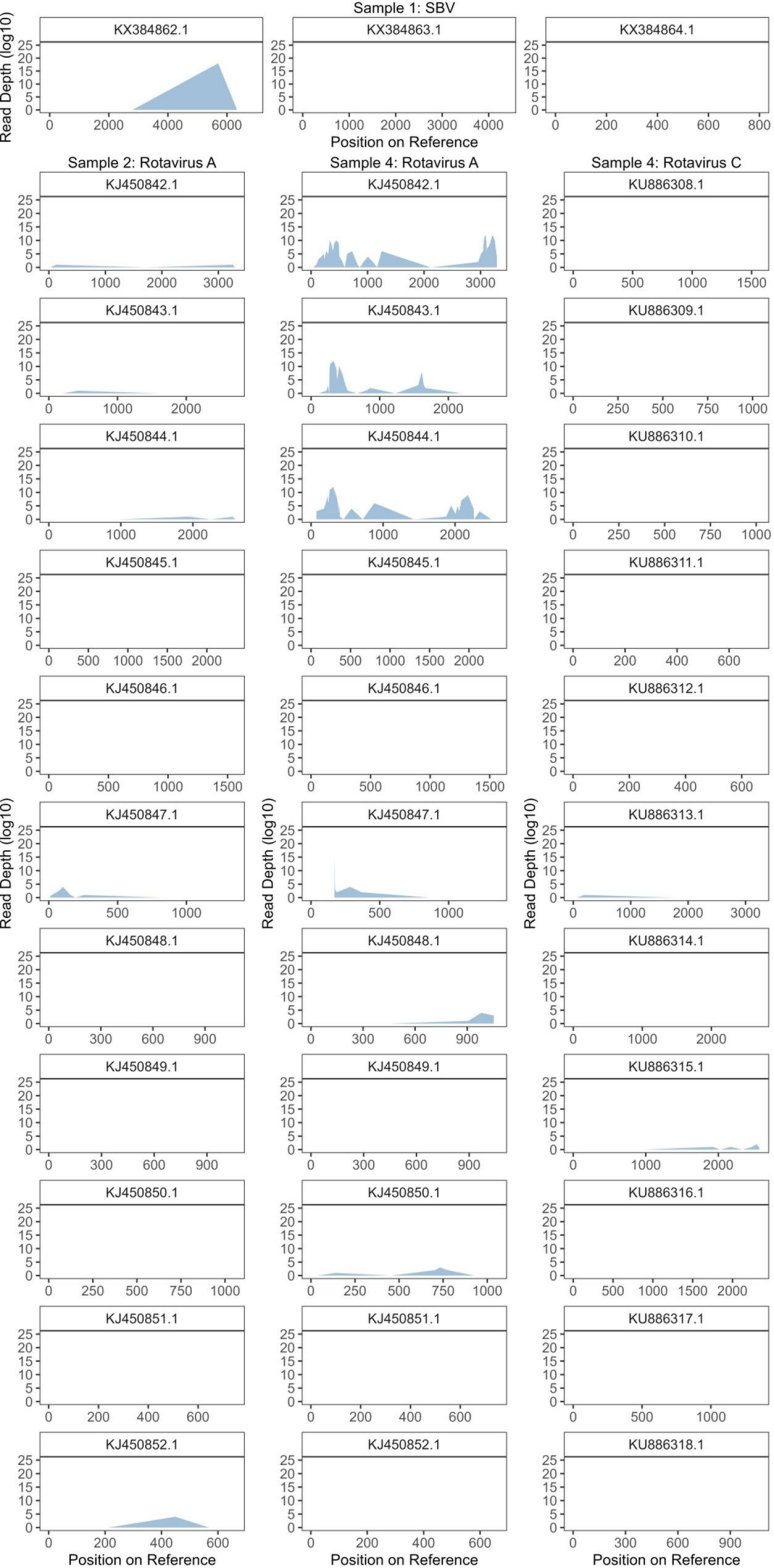
Coverage plots of segmented viruses detected by mNGS. Reads were aligned to references of each segment using Minimap2 and depth of coverage was determined using mosdepth

For the segmented genomes – SBV and Rotavirus – genomes were generated using segment-specific reference sequences (Supplementary Table S7) and coverage plots demonstrated mapping of reads across multiple segments for each Rotavirus (Fig. 4), supporting their classification as true positives. Although reads only mapped to the L segment of SBV, they mapped to multiple loci of this segment (Fig. 4).

Of the known knowns, the greatest coverage and depth was observed for viruses with lower CT values, such as Rotavirus A (CT = 7) in Sample 3, and for samples with fewer freeze-thaw cycles and shorter storage times, such as Mpox IIb from Samples 5 and 7 (Supplementary Figure S3).

#### Reference-based consensus genomes for annotation

**Fig. 5:**
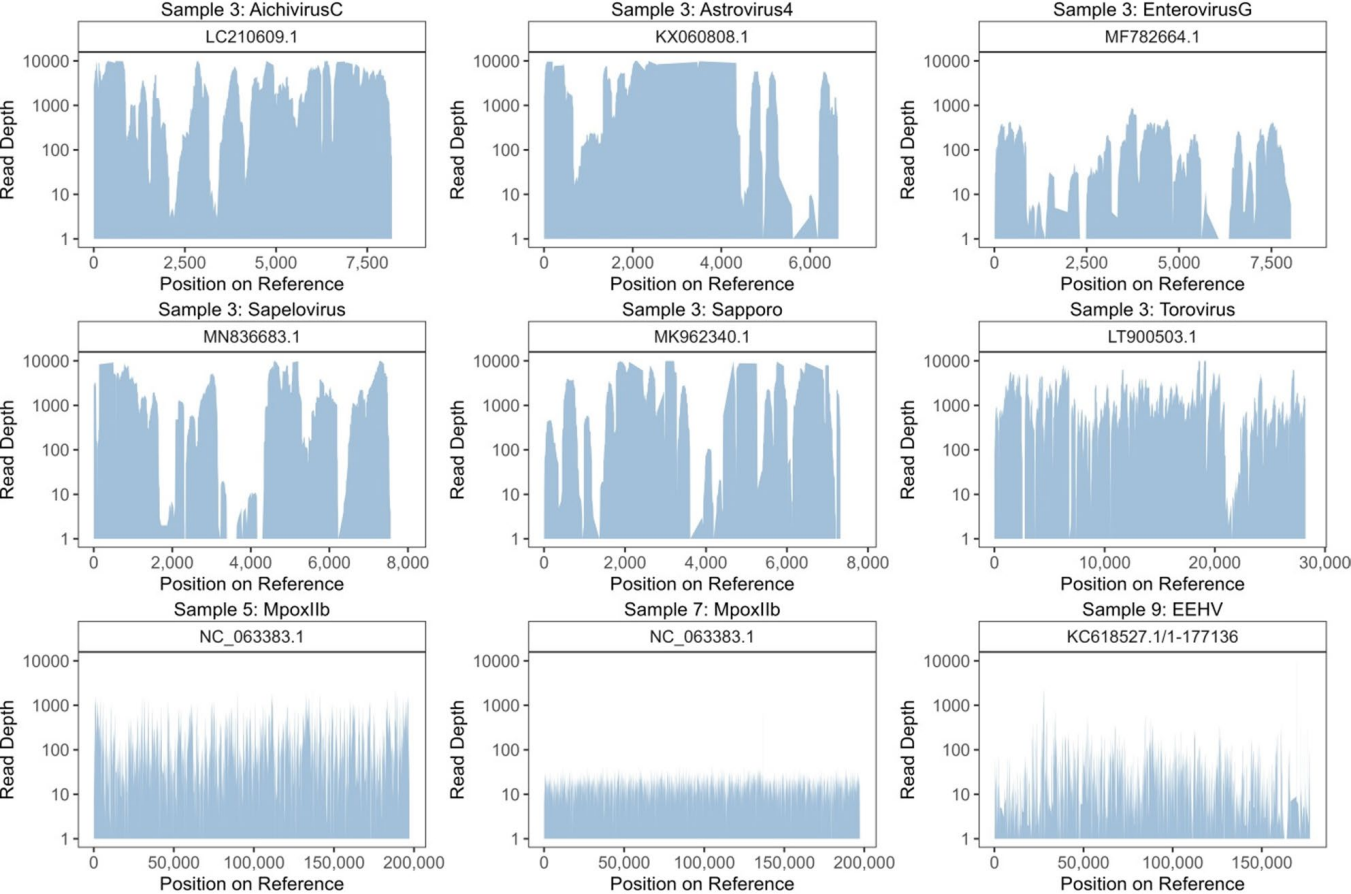
Coverage plots of non-segmented viruses detected by mNGS and with ≥ 75% 1X genome coverage at a mean read depth ≥ 10

High-quality genomes suitable for downstream analyses were those with at least 75% genome coverage at 1x and mean read depth of at least 10 (Table 5) [49]. For nine non-segmented viruses meeting these criteria, coverage plots showed uniformly high depth across the genome length, except Teschovirus A from Sample 3, which had several gaps and areas of low coverage (Fig. 5). All other consensus genomes, which had at least 60% coverage at ≥ 10X read depth (the reduced coverage from 75% to 60% was caused by increased read depth requirements), were annotated with VAPiD [50] and uploaded to GenBank (Supplementary Table 2).

**Fig. 6:**
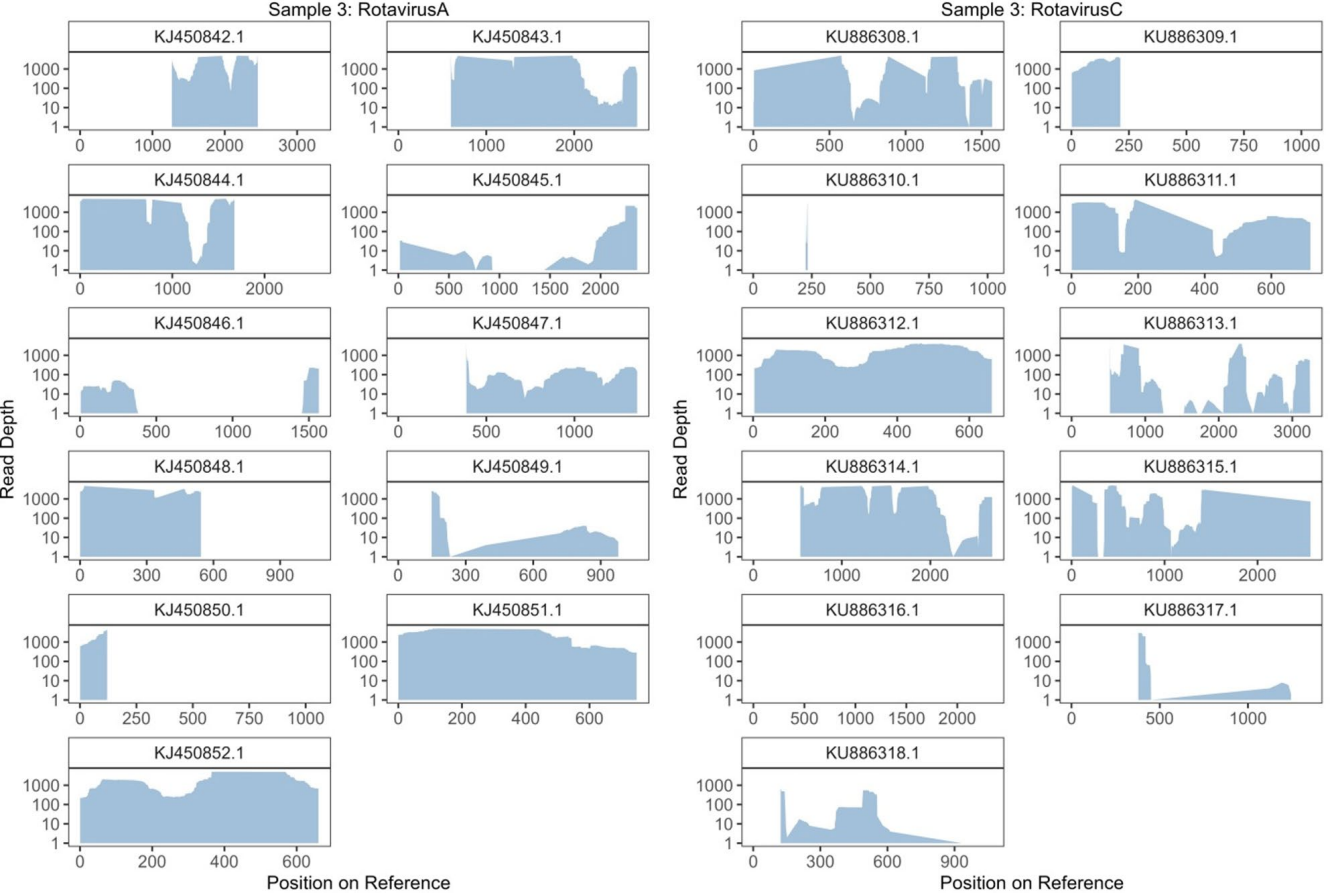
Coverage plots of segmented viruses detected by mNGS and with ≥ 75% 1X genome coverage at a mean read depth ≥ 10

For segmented viruses, coverage plots revealed uneven depth and coverage among segments (Fig. 6). Consensus genomes were annotated with VAPiD [50] and submitted to GenBank for segments with at least 60% coverage at ≥ 10X read depth (Supplementary Table S6).

The frequency and length of N base call stretches, where the depth of coverage was less than 10, was associated with genome length and coverage (Supplementary Table 8). Consensus genomes longer than 30 kb had increased frequency and longer N stretches compared to shorter genomes, while their frequency and length decreased as coverage increased. The failure to detect entire genomic segments meant long N stretches were found for Rotaviruses despite having genomes less than 20 kb.

### Conclusion

Reference-based consensus genomes from mNGS data enabled verification of viral hits from the metagenomics pipeline based on the mapping of reads to multiple loci along reference genomes. High-quality consensus genomes were also generated and deposited on GenBank.

## Discussion

In preparation for future viral outbreaks of unknown aetiology, shotgun mNGS workflows were evaluated using swab and tissue samples positive for a range of known DNA and RNA viruses, collected from multiple anatomical sites and host species and subjected to diverse handling and storage timelines. Detection of 28 known knowns or unknown knowns (co-infections) including segmented, DNA and RNA viral genomes, along with generation of 11 reference-based consensus genomes with at least 60% coverage at greater than 10X depth highlights the power of the workflow to not only identify but also genetically characterise viruses in a range of clinical samples. This level of genetic characterisation is valuable in an outbreak situation as genomic information is vital for the design of diagnostics and vaccines, investigations into virus origin and epidemiology and inference of phenotypic traits [10, 14, 16–22].

QC analyses confirmed that the variety of clinical specimens provided nucleic acid extracts spanning a wide range of concentrations, purity and integrity, yet the workflow remained consistently robust to detect viruses from poor-quality extracts. The QC monitoring included details of sample storage conditions, spectrometric and fluorometric nucleic acid quantification and measurement of nucleic acid integrity. Identification of laboratory and analytical contaminants has highlighted the importance of negative controls to rule out false positives. Despite not being used here, the inclusion of spike-ins for the screening of cases of unknown aetiology would add an extra layer of control as a verification of workflow performance and standardisation between libraries [33].

Applying thresholds of at least two unique matching reads for nucleotide-level searches (NT) and at least 10 unique matching reads for amino acid-level searches (NR), 88.9% of the 18 known viruses were detected, indicating similar performance compared to molecular testing (qPCR and end-point PCR) and demonstrating robustness of the selected extraction, library preparation and sequencing protocols for virus detection by mNGS. As this evaluation encompassed multiple organs, host species, and both DNA and RNA viruses, we adopted relatively low, matrix agnostic minimum read thresholds and required additional supporting evidence (confirmatory BLAST, non-overlapping loci and genome coverage) to balance sensitivity and specificity. Similar low read thresholds combined with multi parameter criteria have been used in other clinical mNGS assays applied to heterogeneous specimen types [46–48]. In this exploratory study, these thresholds should be regarded as provisional operating criteria rather than definitive cut-offs and are explicitly recognised as a limitation that will require refinement in larger cohorts.

The two-read NT threshold was supported by identification of four previously detected viruses in samples 2, 4, 8, and 13 with only two reads (Table 3), with these hits further corroborated by the increased mapping during reference-based consensus genome generation (Table 5). Application of a higher read threshold would have resulted in false-negative results driven by sample conditions, which likely reflect those in a potential outbreak setting including diverse matrix types, variable storage conditions with multiple freeze-thaw cycles, different viral genetic material, viral genome fragmentation and size. In an outbreak scenario, failure to report such low level but genuine detections could have adverse welfare and economic consequences. Importantly, putative low read hits without additional supporting evidence would be considered low-confidence positives and such putative hits would not be acted upon directly, but instead would trigger further evaluation using clinical, epidemiological and orthogonal molecular tests such as targeted PCR.

As only one virus lacking a reference genome was included, the 10-read NR threshold was selected arbitrarily and currently lacks supporting data. This NR threshold is therefore also provisional and will be revised as additional viruses without close references are interrogated in larger sample sets.

In terms of sensitivity of mNGS relative to RT-qPCR, the workflows detected all viruses with CTs less than 30 with the two unidentified viruses having CTs of 30 and 31. Along with virus quantity, the ability to detect viruses by mNGS is also influenced by storage conditions that effect nucleic acid integrity; anatomical site that influences the quantity of background host nucleic acid; and host that effects rRNA depletion efficiency. As larger cohorts of related clinical specimens are analysed, more definitive CT limits of detection can be established for different sample types.

Detection of previously unrecognised or poorly characterised viruses is a key requirement of any mNGS-based pathogen surveillance system. The workflows established here generated data of sufficient quality to identify additional viruses in samples not previously tested for, illustrating the capacity to detect previously unrecognised co-infections (“unknown knowns”). In addition, members of the *Gammaherpesvirus* genus were identified in Sample 14 using an amino acid search of the NR database, consistent with the presence of PHV7 – a virus lacking a complete reference genome in the NT database. The logical next step is to apply these mNGS workflows to cases of suspected infectious disease of unknown aetiology, to assess performance in identifying truly novel or unanticipated viral infection, including unknown unknowns [55].

Reference-based consensus genomes were used to verify metagenomic hits by examining how reads distributed along reference genomes, enabling the identification of potential false positives. Identification of potential false positives in this study highlights the importance of complementing automated metagenomic classification with an independent verification step or other orthogonal methods, such as PCR or consensus genome generation.

The main limitation of this study is the diverse but small sample size, which limited the scope for statistical validation of the workflows but enabled testing of their robustness. Questions also remain over workflow reproducibility as technical replicates were not included and inter-run variability was not tested. Inclusion of spike-ins to standardise libraries across samples and act as positive controls will strengthen the workflows when screening samples of unknown aetiology. Despite identification of PHV7 with as little as 45% amino acid identity, detection of viruses sharing less homology requires testing because there is potential for such viruses to emerge in animal or human populations. For assurances over their suitability for pandemic preparedness, workflows require testing with larger cohorts for statistical measures of their sensitivity, specificity and reproducibility, along with more diverse samples to test their capacity for detection of truly novel viruses lacking amino acid level homology with viruses in NR databases.

Focussing on the tissue workflow, the effectiveness of the transcriptomics approach for detection of latent viruses was not assessed. The lower transcriptomic activity of latent viruses could result in viral transcripts below the limit of detection of the mNGS tissue workflow. Failure to detect latent viruses could prevent detection of pathogens causing potentially serious long-term sequalae.

Previous studies have described mNGS workflows for viral pathogen identification across multiple sample matrices, hosts and anatomical sites [27, 29, 34, 36, 37, 54, 56, 57]. The present study extends this by testing a shotgun mNGS workflow on clinical samples from multiple anatomical sites and host species, while explicitly documenting its robustness across nucleic acid extracts of varying concentrations, purity and integrity. Collectively, the tissue and swab mNGS workflows developed here were effective at detecting known viruses, previously unrecognised infections (unknown knowns), and one partially characterised agent (known unknowns) from a spectrum of clinically relevant samples. This has demonstrated the feasibility of these workflows for integration into passive and syndromic surveillance networks where their performance in real-world investigations of suspected infectious disease cases of unknown aetiology can be truly evaluated.

## Supplementary Information

Supplementary Material 1.

## Data Availability

The datasets generated during the current study are available in the Sequence Read Archive repository, [https://www.ncbi.nlm.nih.gov/sra/PRJNA1371775] Annotated complete/partial genomes and contigs have been uploaded to Genbank (full list of accession numbers in Supplementary Table 2).
