# Supplementary material for: One health viral metagenomics for pathogen surveillance: robust mNGS workflows for viral detection and genome recovery from swab and tissue specimens
Source: BMC Microbiol. 2026 May 29;26:688. doi: 10.1186/s12866-026-05105-5 (PMC13435701; doi:10.1186/s12866-026-05105-5)
Supplement: Supplementary file 1 [file 12866_2026_5105_MOESM1_ESM.docx]

**Supplementary Information**

| **Sample** | **Host** | **Body Location** | **Matrix** | **BioSample Accessions** | |
| --- | --- | --- | --- | --- | --- |
|  |  |  |  | **DNA Library** | **RNA Library** |
| 1 | *Bos taurus* | Abomasal Fluid | Swab | SRR36250585 | SRR36250584 |
| 2 | *Sus scrofa* | Small Intestine | Swab | SRR36250583 | SRR36250582 |
| 3 | *Sus scrofa* | Large Intestine | Swab | SRR36250594 | SRR36250593 |
| 4 | *Sus scrofa* | Faecal | Swab | SRR36250592 | SRR36250591 |
| 8 | *Gallus gallus* | Trachea | Swab | SRR36250590 | NA |
| 9 | *Elephas maximus* | Heart | Tissue | NA | SRR36250596 |
| 10 | *Cervus nippon* | Liver | Tissue | NA | SRR36250595 |
| 11 | *Columba livia* | Brain | Tissue | NA | SRR36250586 |
| 12 | *Gallus gallus* | Intestine | Tissue | NA | SRR36250588 |
| 13 | *Halichoerus grypus* | Brain | Tissue | NA | SRR36250589 |
| 14 | *Halichoerus grypus* | Gingiva | Tissue | NA | SRR36250587 |

**Supplementary Table S1:** *Sequence Read Archive BioSample Accessions for raw fastq files produced in this study. Only non-human fastq files were submitted to Sequence Read Archive under BioProject PRJNA1371775.*

| Sample | Virus | GenBank Accession(s) |
| --- | --- | --- |
| 3 | Rotavirus A | PX848832-PX848840 |
|  | Rotavirus C | PX848841- PX848848 |
|  | Sapporo virus | PX848850 |
|  | Sapelovirus | PX848851 |
|  | Aichivirus C | PX848852 |
|  | Astrovirus 4 | PX848849 |
|  | Porcine torovirus | PX848853 |
|  | Enterovirus G | PX848854 |
| 5 | Mpox IIb | PX655238 |
| 7 | Mpox IIb | PX655239 |
| 9 | EEHV | PX848831 |
| 14 | PHV7 (contigs) | PX848855-PX848913 |

**Supplementary Table S2:** GenBank accessions for assembled and annotated genomes and PHV7 contigs.

| **Sample** | **Known Virus** | **Detection by PCR** | **Freeze-Thaw Cycles** | **Storage (°C)** | **Time in Storage** | **Detection by mNGS** |
| --- | --- | --- | --- | --- | --- | --- |
| 1 | SBV | qPCR (CT=32) | 1 | -20 & -80 | 1 month | Yes |
| 2 | Rotavirus A | qPCR (CT=27) | ≥1 | -80 | 10 years | Yes |
|  | Rotavirus B | qPCR (CT=31) |  |  |  | No |
| 3 | Rotavirus A | qPCR (CT=7) | ≥1 | -80 | 10 years | Yes |
|  | Rotavirus B | qPCR (CT=17) |  |  |  | Yes |
|  | Rotavirus C | qPCR (CT=15) |  |  |  | Yes |
| 4 | Rotavirus A | qPCR (CT=23) | ≥1 | -80 | 10 years | Yes |
|  | Rotavirus C | qPCR (CT=22) |  |  |  | Yes |
| 5 | Mpox IIb | qPCR (CT=27) | 0 | Processed on arrival | 0 days | Yes |
| 6 | Mpox Ia | qPCR (CT=24) | 0 | Processed on arrival | 0 days | Yes |
| 7 | Mpox IIb | qPCR (CT=) | 0 | Processed on arrival | 0 days | Yes |
| 8 | MDV | qPCR (CT=33) | ≥1 | -80 | 1 year | Yes |
| 9 | EEHV | PCR + | 1 | -20 & -80 | 1 month | Yes |
| 10 | OHV2 | PCR + | 1 | -20 & -80 | 1 month | Yes |
| 11 | PMV1 | qPCR (CT=30) | ≥1 | -80 | 4 years | No |
| 12 | IBV | qPCR (CT=26) | ≥1 | -80 | 8 months | Yes |
| 13 | PHV1 | PCR + | 2 | -20 & -80^1^ | 2 years | Yes |
| 14 | PHV7 | PCR + | 2 | -20 & -80^1^ | 6 months | Yes |

**Supplementary Table S3:** *Sample handling and storage conditions and qualitative detection by mNGS.*

| **Sample** | **Known Virus** | **Detection by PCR** | **Qubit Concentration (ng/µl)** | | **Nanodrop Purity Ratios** | | **Bioanalyzer** | | **Detection by mNGS** |
| --- | --- | --- | --- | --- | --- | --- | --- | --- | --- |
|  |  |  | **DNA** | **RNA** | **260/280** | **260/230** | **RIN** | **DV200** |  |
| 1 | SBV | qPCR (CT=32) | 0.113 | <LOD | 3.68 | 0.13 | NA | NA | Yes |
| 2 | Rotavirus A | qPCR (CT=27) | <LOD | <LOD | 1.37 | 0.61 | NA | NA | Yes |
|  | Rotavirus B | qPCR (CT=31) |  |  |  |  |  |  | No |
| 3 | Rotavirus A | qPCR (CT=7) | 0.562 | <LOD | 1.52 | 0.79 | 2.3 | >70% | Yes |
|  | Rotavirus B | qPCR (CT=17) |  |  |  |  |  |  | Yes |
|  | Rotavirus C | qPCR (CT=15) |  |  |  |  |  |  | Yes |
| 4 | Rotavirus A | qPCR (CT=23) | 0.521 | 18.3 | 2.06 | 1.55 | NA | NA | Yes |
|  | Rotavirus C | qPCR (CT=22) |  |  |  |  |  |  | Yes |
| 5 | Mpox IIb | qPCR (CT=27) | <LOD | < LOD | 1.96 | 0.73 | NA | NA | Yes |
| 6 | Mpox Ia | qPCR (CT=24) | 0.662 | 1.35 | 1.93 | 0.73 | NA | NA | Yes |
| 7 | Mpox IIb | qPCR (CT=) | 49.0 | <LOD | 1.99 | 1.82 | NA | NA | Yes |
| 8 | MDV | qPCR (CT=33) | 0.055 | <LOD | 1.01 | 0.67 | NA | NA | Yes |
| 9 | EEHV | PCR + | NA | 72.9 | 1.97 | 1.87 | 2.1 | 50-70% | Yes |
| 10 | OHV2 | PCR + | NA | 569.4 | 2.01 | 2.03 | 6.8 | NA | Yes |
| 11 | PMV1 | qPCR (CT=30) | NA | 36.0 | 1.78 | 1.32 | 1.2 | >70% | No |
| 12 | IBV | qPCR (CT=26) | NA | 54.1 | 1.85 | 1.14 | 7.7 | NA | Yes |
| 13 | PHV1 | PCR + | NA | 143.1 | 2.04 | 2.00 | 5.3 | NA | Yes |
| 14 | PHV7 | PCR + | NA | 6.3 | 3.17 | 0.67 | NA | 30-50% | Yes |

**Supplementary Table S4:** *Nucleic acid extract measurements and qualitative detection by mNGS. RNA integrity score (RIN) measured RNA integrity based on detection of 18S and 28S rRNA with a higher score indicating increased integrity. DV200 is the proportion of RNA fragments >200 bp.*

|  | **%Q30** | **Yield (GB)** | **Reads Passing Filter** | **Occupancy** |
| --- | --- | --- | --- | --- |
| Illumina Specifications | >85% | 120 | 499,000,000 | NA |
| Run 1 (Tissues) | 90.66% | 166.55 | 530,503,616 (79.66%) | 99.63% |
| Run 2 (Swabs) | 88.39% | 158.27 | 505,791,936 (75.95%) | 99.81% |
| Run 3 (Swabs/Bloods) | 88.68% | 162.74 | 518,776,524 (77.81%) | 99.86% |

**Supplementary Table S5:** *Illumina NextSeq 2000 run parameters. %>Q30 is the percentage of base calls made with high confidence applying a cutoff of 30. Yield is the amount of data generated. Reads passing filter is the number and percentage of clusters, which could be read by the instrument with a low count or percentage indicative of under or over clustering, respectively. Occupancy is the number of cites occupied with library on the flow cell.*

| **Library** | **Virus** | **Reads (DB)** | **Detection in Other Samples (Reads)** |
| --- | --- | --- | --- |
| DNA | Syrbvirus sp. | 12 (NT) | None |
| DNA | Lentivirus/HIV | 10 (NT) | S1 (10), S13 (2) |
| DNA | Betapapillomavirus 2 | 8 (NT) | S5 (16) |
| DNA and RNA | Picobirnavirus sp. | 16 (NT and NR) | S2 (104), S3 (33), S4 (1), S5 (311), S6 (6), S8 (4), S9 (4), S11 (991), S12 (9), S14 (144) |

**Supplementary Table S6:** Viruses detected in the negative control DNA and RNA libraries. A water control was included throughout the metagenomics workflow and used to detect contaminants from the laboratory setting.

**Supplementary Figure S1:** *Gross pathological examination of a haemorrhagic Asian Elephant. A) Gross pathological images of the heart and intestine. Cardiac petechial and ecchymotic haemorrhages were observed in the heart and ulceration in the intestine. These observations were used in selecting heart tissue for mNGS analysis.*

**Supplementary Figure S2:** *Binary coverage plot for PHV7. A consensus sequence was generated by selecting a suitable reference from the matches in the metagenomic pipeline. A BLASTX search was then performed with Diamond to identify the coordinates of matching reads. These were then reverse translated and mapped back to the reference genome.*

| **Sample** | **Virus** | **Library** | **Ref. Accession** | **Length** | **No. of Reads** | **1X Coverage (%)** | **Mean Depth** |
| --- | --- | --- | --- | --- | --- | --- | --- |
| 1 | SBV | cDNA | KX384862.1 | 6882 | 36 | 2.9 | 0.78 |
|  |  |  | KX384864.1 | 798 | 0 | 0 | 0 |
|  |  |  | KX384863.1 | 4373 | 0 | 0 | 0 |
| 2 | Rotavirus A | cDNA | KJ450842.1 | 3302 | 2 | 5.7 | 0.057 |
|  |  |  | KJ450843.1 | 2717 | 1 | 2.1 | 0.021 |
|  |  |  | KJ450844.1 | 2591 | 2 | 4.8 | 0.048 |
|  |  |  | KJ450847.1 | 1356 | 5 | 16.6 | 0.30 |
|  |  |  | KJ450852.1 | 662 | 8 | 7.9 | 0.60 |
|  |  |  | KJ450845.1 | 2362 | 0 | 0 | 0 |
|  |  |  | KJ450846.1 | 1566 | 0 | 0 | 0 |
|  |  |  | KJ450848.1 | 1075 | 0 | 0 | 0 |
|  |  |  | KJ450850.1 | 1059 | 0 | 0 | 0 |
|  |  |  | KJ450849.1 | 1062 | 0 | 0 | 0 |
|  |  |  | KJ450851.1 | 750 | 0 | 0 | 0 |
| 3 | Rotavirus A | cDNA | KJ450842.1 | 3302 | 6090962 | 100 | 199449 |
|  |  |  | KJ450843.1 | 2717 | 4161241 | 100 | 138441 |
|  |  |  | KJ450844.1 | 2591 | 4436893 | 100 | 205067 |
|  |  |  | KJ450845.1 | 2362 | 3134 | 52.4 | 155.1 |
|  |  |  | KJ450846.1 | 1566 | 414 | 34.5 | 21.4 |
|  |  |  | KJ450847.1 | 1356 | 7053501 | 100 | 197494 |
|  |  |  | KJ450848.1 | 1075 | 465633 | 100 | 62142.1 |
|  |  |  | KJ450850.1 | 1059 | 2425163 | 100 | 308578 |
|  |  |  | KJ450849.1 | 1062 | 88600 | 51.7 | 6906.3 |
|  |  |  | KJ450851.1 | 750 | 154339 | 100 | 28281.3 |
|  |  |  | KJ450852.1 | 662 | 36506 | 100 | 7406.2 |
| 3 | Rotavirus A | DNA | KJ450842.1 | 3302 | 51 | 39.7 | 1.8 |
|  |  |  | KJ450843.1 | 2717 | 1 | 3.8 | 0.038 |
|  |  |  | KJ450844.1 | 2591 | 50 | 28.1 | 2.08 |
|  |  |  | KJ450847.1 | 1356 | 21 | 34.9 | 2.04 |
|  |  |  | KJ450850.1 | 1059 | 4 | 19.6 | 0.49 |
|  |  |  | KJ450851.1 | 750 | 4 | 28.3 | 0.56 |
|  |  |  | KJ450852.1 | 662 | 2 | 6.2 | 0.12 |
|  |  |  | KJ450845.1 | 2362 | 0 | 0 | 0 |
|  |  |  | KJ450846.1 | 1566 | 0 | 0 | 0 |
|  |  |  | KJ450848.1 | 1075 | 0 | 0 | 0 |
|  |  |  | KJ450849.1 | 1062 | 0 | 0 | 0 |
| 3 | Rotavirus B | cDNA | KR052714.1 | 3512 | 34 | 3.8 | 0.66 |
|  |  |  | KR052716.1 | 2342 | 10 | 17.3 | 0.55 |
|  |  |  | KR052712.1 | 750 | 318 | 23.7 | 53.6 |
|  |  |  | KR052715.1 | 2843 | 0 | 0 | 0 |
|  |  |  | KR052717.1 | 2300 | 0 | 0 | 0 |
|  |  |  | KR052709.1 | 1277 | 0 | 0 | 0 |
|  |  |  | KR052718.1 | 1268 | 0 | 0 | 0 |
|  |  |  | KR052711.1 | 978 | 0 | 0 | 0 |
|  |  |  | KR052710.1 | 1030 | 0 | 0 | 0 |
|  |  |  | KR052719.1 | 811 | 0 | 0 | 0 |
|  |  |  | KR052713.1 | 643 | 0 | 0 | 0 |
| 3 | Rotavirus C | cDNA | KU886313.1 | 3248 | 3719221 | 91.2 | 85535.9 |
|  |  |  | KU886314.1 | 2701 | 1581179 | 98.6 | 74471.5 |
|  |  |  | KU886315.1 | 2571 | 4888329 | 98.1 | 165443 |
|  |  |  | KU886316.1 | 2343 | 380 | 1.5 | 5.51344 |
|  |  |  | KU886308.1 | 1569 | 330532 | 99.0 | 27676.6 |
|  |  |  | KU886317.1 | 1355 | 6439515 | 45.8 | 142562 |
|  |  |  | KU886310.1 | 1020 | 3539184 | 100 | 296422 |
|  |  |  | KU886309.1 | 1040 | 1777574 | 100 | 231641 |
|  |  |  | KU886318.1 | 1069 | 86336 | 51.2 | 8453.9 |
|  |  |  | KU886311.1 | 718 | 70939 | 100 | 12954.7 |
|  |  |  | KU886312.1 | 664 | 12815 | 100 | 2622.02 |
| 4 | Rotavirus A | DNA | KJ450842.1 | 3302 | 90 | 41.7 | 3.5 |
|  |  |  | KJ450843.1 | 2717 | 52 | 22.9 | 2.1 |
|  |  |  | KJ450844.1 | 2591 | 62 | 50.1 | 3.3 |
|  |  |  | KJ450847.1 | 1356 | 123 | 27.1 | 13.5 |
|  |  |  | KJ450848.1 | 1075 | 7 | 18.0 | 0.93 |
|  |  |  | KJ450850.1 | 1059 | 7 | 23.3 | 0.7 |
|  |  |  | KJ450845.1 | 2362 | 0 | 0 | 0 |
|  |  |  | KJ450846.1 | 1566 | 0 | 0 | 0 |
|  |  |  | KJ450849.1 | 1062 | 0 | 0 | 0 |
|  |  |  | KJ450851.1 | 750 | 0 | 0 | 0 |
|  |  |  | KJ450852.1 | 662 | 0 | 0 | 0 |
| 4 | Rotavirus C | cDNA | KU886313.1 | 3248 | 1 | 4.2 | 0.041 |
|  |  |  | KU886315.1 | 2571 | 4 | 12.5 | 0.15 |
|  |  |  | KU886314.1 | 2701 | 0 | 0 | 0 |
|  |  |  | KU886316.1 | 2343 | 0 | 0 | 0 |
|  |  |  | KU886308.1 | 1569 | 0 | 0 | 0 |
|  |  |  | KU886317.1 | 1355 | 0 | 0 | 0 |
|  |  |  | KU886310.1 | 1020 | 0 | 0 | 0 |
|  |  |  | KU886309.1 | 1040 | 0 | 0 | 0 |
|  |  |  | KU886318.1 | 1069 | 0 | 0 | 0 |
|  |  |  | KU886311.1 | 718 | 0 | 0 | 0 |
|  |  |  | KU886312.1 | 664 | 0 | 0 | 0 |

**Supplementary Table S7:** *Breakdown of consensus sequence metrics for each segment of SBV and Rotavirus species.*

**Supplementary Figure S3:** Correlation between CT and genome coverage (A) and mean read depth (B) along with their relationship to freeze-thaw cycles.

| **Sample** | **Virus** | **Genome Length (bp)** | **Coverage at 10X (%)** | **Stretches of N** | **Longest Stretch (bp)** | **Mean Length (bp)** | **Length of N Stretches** | | | |
| --- | --- | --- | --- | --- | --- | --- | --- | --- | --- | --- |
|  |  |  |  |  |  |  | **1-10 (bp)** | **11-100 (bp)** | **101-1000 (bp)** | **>1001 (bp)** |
| 3 | Aichivirus C | 8183 | 95.7 | 4 | 200 | 88 | 2 | 0 | 2 | 0 |
|  | Porcine astrovirus 4 | 6644 | 86.4 | 5 | 679 | 180.2 | 0 | 3 | 2 | 0 |
|  | Enterovirus G | 8072 | 71.5 | 11 | 686 | 209.2 | 0 | 5 | 6 | 0 |
|  | Sapelovirus A | 7560 | 80.7 | 10 | 583 | 145.9 | 2 | 4 | 4 | 0 |
|  | Sapporo virus | 7320 | 87.1 | 11 | 337 | 85.9 | 3 | 4 | 4 | 0 |
|  | Porcine torovirus | 28,224 | 95.4 | 15 | 519 | 87.4 | 3 | 8 | 4 | 0 |
|  | Rotavirus A | 18,502 | 81.9 | 11 | 1110 | 304.6 | 2 | 3 | 5 | 1 |
|  | Rotavirus C | 18,298 | 70.5 | 21 | 2309 | 257 | 4 | 8 | 8 | 1 |
| 5 | Mpox IIb | 197,200 | 89.7 | 215 | 598 | 94.7 | 33 | 107 | 75 | 0 |
| 7 | Mpox IIb | 197,215 | 94.4 | 398 | 355 | 27.7 | 162 | 213 | 23 | 0 |
| 9 | EEHV1A | 177,162 | 63.5 | 416 | 3289 | 155.4 | 103 | 157 | 149 | 7 |

**Supplementary Table S8:** Breakdown of N base call stretches in reference-assembled consensus genomes with 10X depth.
